# Field-omics—understanding large-scale molecular data from field crops

**DOI:** 10.3389/fpls.2014.00286

**Published:** 2014-06-20

**Authors:** Erik Alexandersson, Dan Jacobson, Melané A. Vivier, Wolfram Weckwerth, Erik Andreasson

**Affiliations:** ^1^Department of Plant Protection Biology, Swedish University of Agricultural SciencesAlnarp, Sweden; ^2^Department of Viticulture and Oenology, Institute for Wine Biotechnology, Stellenbosch UniversityStellenbosch, South Africa; ^3^Department of Ecogenomics and Systems Biology, University of ViennaVienna, Austria

**Keywords:** field trials, crops, transcriptomics, proteomics, metabolomics, bioinformatics, field sampling, field-omics

## Abstract

The recent advances in gene expression analysis as well as protein and metabolite quantification enable genome-scale capturing of complex biological processes at the molecular level in crop field trials. This opens up new possibilities for understanding the molecular and environmental complexity of field-based systems and thus shedding light on the black box between genotype and environment, which in agriculture always is influenced by a multi-stress environment and includes management interventions. Nevertheless, combining different types of data obtained from the field and making biological sense out of large datasets remain challenging. Here we highlight the need to create a cross-disciplinary platform for innovative experimental design, sampling and subsequent analysis of large-scale molecular data obtained in field trials. For these reasons we put forward the term field-omics: “Field-omics strives to couple information from genomes, transcriptomes, proteomes, metabolomes and metagenomes to the long-established practice in crop science of conducting field trials as well as to adapt current strategies for recording and analysing field data to facilitate integration with ‘-omics’ data.”

## Introduction

In the nineteenth century agricultural field experiments became widely practiced and have since been a central part of crop science. These trials have on several occasions driven scientific discovery forward and given valuable insights on, for example, the effects of fertilization and crop-rotation on yield or the transmission of plant pathogens. The results have been applied both in extension services and environmental regulations (Alm, 2007). They have also formed the basis for conceptual frameworks such as Ronald Fisher's pioneering work on the theory of experimental design driven by the extensive collection of field data, which paved the way for new statistical methods. Traditionally, external parameters for weather and soil composition have been recorded in field trials whereas assessment of crop performance has been limited to external measurements of variables such as disease progression and yield, or limited analysis of levels of amino acids, sugars and nutrients often related to crop quality.

The recent flurry of technological advancements by, e.g., Next-Generation sequencing (NGS) and mass spectrometry (MS), have enabled genome-scale capturing of biological processes at the molecular level (Weckwerth, 2011). Rather than being limited to a handful of measured compounds, it is now possible to capture thousands of molecular variables. However, these techniques have mostly been applied in the laboratory and controlled environments. The generation of such large datasets—often referred to as “-omics” data—demands partly new considerations for experimental set-ups, sampling, data analysis and visualization. Combining different types of data and making sense of large datasets remains challenging and, so far, relatively few studies have related molecular processes to environmental factors in the field and the effects of a multi-stress environment remain poorly studied.

It is well-known that developmental and morphological differences caused by the artificial environment imposed in a laboratory setting can mask important crop traits. For example, a saline hydroponic lab set-up could not reliably predict differences in salt tolerance between barley cultivars when subsequently grown in natural saline soils (Tavakkoli et al., 2012) and a meta-analysis found contradicting results between studies estimating crop responses to herbicides when done in greenhouse vs. field settings (Clark et al., 2004). Strikingly, the growth-data variance between the different settings was on the same level as that found from entirely different species within the same genus.

Light in field conditions have a large impact on plant growth. For example, outdoors the model plant Arabidopsis displays changed leaf morphology with altered pigment composition and fitness performance (Külheim et al., 2002; Mishra et al., 2012). This can be ascribed to rapidly changing outdoor light intensities that influence genes in photo-oxidative stress. Natural light conditions also influence the infection success of pathogens (reviewed in Kangasjarvi et al., 2012), which is important to consider since plant-pathogen interactions in the laboratory mostly are conducted in relatively low light intensity.

Field resistance is something only observed in a field situation but not in the laboratory. It is assumed to be controlled by more than a single genetic locus and subject to environmental cues. For example, in the late blight-resistant potato cultivar, Sarpo Mira, a discrepancy between resistance in laboratory tests and field trials was discovered and later linked to an additional resistant factor conferring field resistance (Rietman et al., 2012). These selected examples illustrate the importance of moving from a laboratory to a field setting to understand phenomena only observed there.

**Figure 1:**
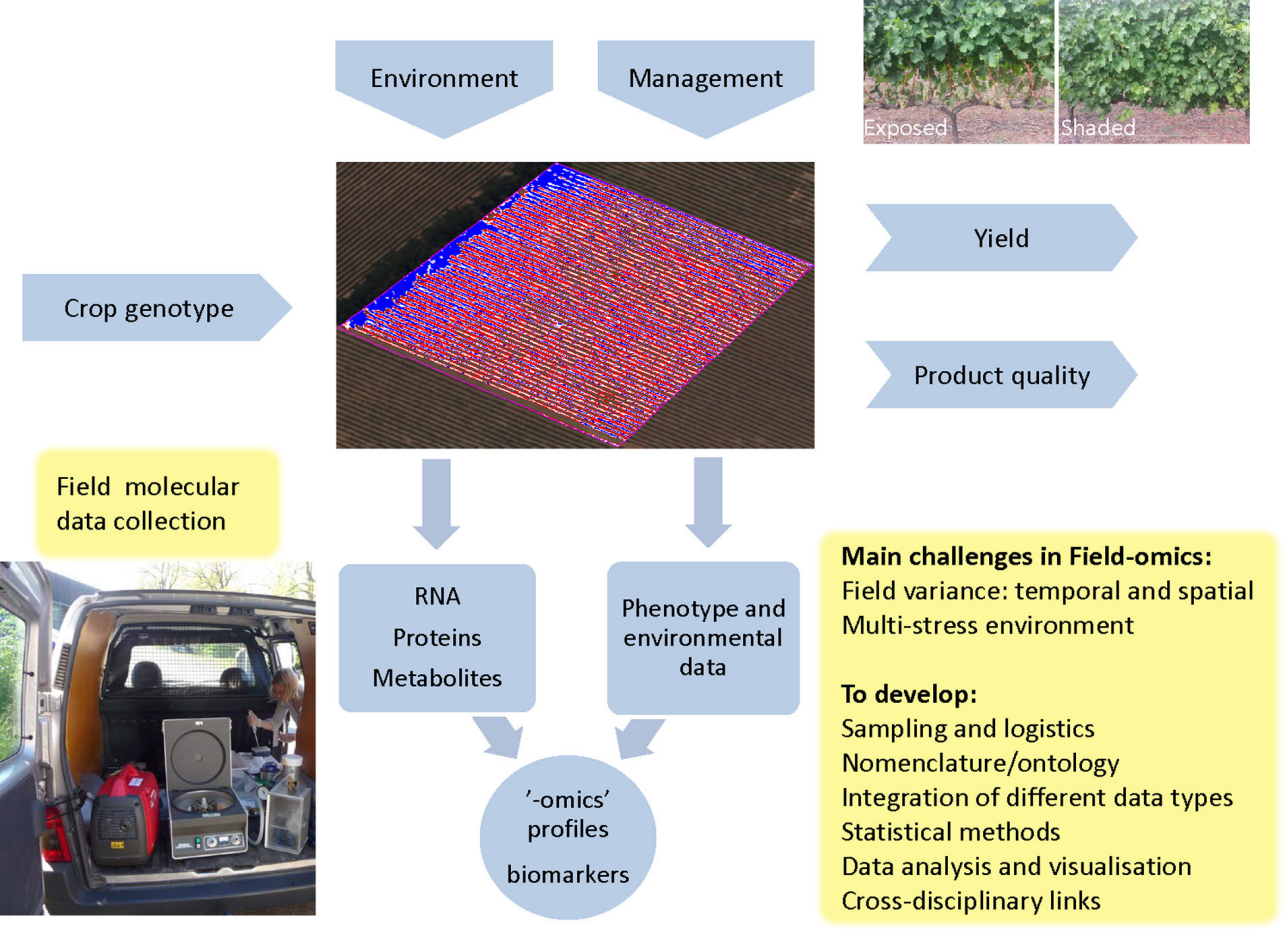
**Field-omics: context and main challenges**. Crop genotypes are influenced by the management practices employed as well as the multi-stress and multi-organism environment present in an agricultural field, which is the central entity in Field-omics. Here the field is illustrated by a multispectral image to visualize the relative vigor of individual vines in a vineyard, which can be one of many considerations for plot layouts and sampling design. A combination of external factors gives rise to a crop phenotype which in turn determines yield and quality. One type of management practice, which has been explored by genome-wide transcriptomics, is the shading or exposure of grape bunches as illustrated in the top right image (Young et al., 2012; Photo: Zelmari Coetzee). A key element in Field-omics is the collection of multivariate molecular data, as shown in the figure where a mobile lab is used for high-throughput, on site isolation of plant secretome samples for proteomic analysis (Dr Åsa Lankinen as photographed by Erik Andreasson). Note that the gene-gun equipment seen in the picture is used for efficient vacuum-infiltration of leaves. “-omic” profiles can subsequently be analyzed in conjunction with phenotypic traits and the environmental factors measured in the field. From this one can identify robust sets of biomarkers for biological processes, pathogens and stress conditions. These can then be used in breeding programs or for the creation of decision support systems for management intervention. “-omic” profiles can also be used for Crop Systems Biology (CBS). As indicated in the boxed text, a number of key issues have to be addressed in order to create a Field-omics platform.

The interaction between genotype and environment on phenotype has been reviewed extensively and the advantages of systems-biology-based approaches have been proposed (Keurentjes et al., 2011; Weckwerth, 2011). Here, we want to draw attention to the importance of recording and analyzing different types of molecular data obtained in field trials and relate it to other factors, such as environment and phenotype, and how exploration of these relationships can be used for management intervention and molecular-level discovery (Figure 1 gives a general overview and future challenges). Sampling design and data collection need careful attention in order to capture representative high-dimension datasets for meaningful systems biology in a field setting. If done properly, it can form a cornerstone in the previously coined concept of Crop Systems Biology, which includes modeling of complex crop traits by combining functional genomics with crop physiology and biochemistry (Yin and Struik, 2007). Table 1 outlines certain aspects of Field-omics in relation to other areas.

**Table 1:** **Nine aspects highlighting the similarities and differences between field-omics and the emerging areas of Crop Systems Biology (CSB) and phenomics as well as the established areas of plant molecular lab and ecological field studies**.

	**Field-omics**	**CSB**	**Phenomics**	**Plant molecular lab**	**Ecological field studies**
Experimental setting	Agricultural fields	Agricultural fields or lab	Agricultural fields or lab	Lab	Natural fields
Main study focus	“-omics” profiles in agricultural systems	Creating models for agricultural systems	Measuring multiple traits	Molecular functions	Populations and evolution
#Variables measured	100–10,000 s	100–10,000 s	5–100	1–10,000 s	Ca 10
Number of environmental factors	Medium	High-low	High-low	Low	High
Agricultural management interventions	Yes	Yes	Yes/No	No	No
Statistical analyses	In development	In development	In development	Established	Established
Modeling	No	Yes	Yes	Yes/No	Yes
Biomarker discovery	Yes, molecular	Yes, molecular	Yes, phenotypical	Yes, but robustness?	No
Cross-disciplinary	High	High	High-medium	Low	Low

## “-Omics” techniques for field studies

Genome-wide transcriptome analysis of field samples has highlighted the high variance caused by spatial and temporal differences in the field and identified both plastic and non-plastic genes (e.g., Bläsing et al., 2005; Lund et al., 2008; Dal Santo et al., 2013), a fact that was previously overlooked. NGS-techniques make it feasible to study organisms with an un-sequenced genome as well as different cultivars. In addition, it enables meta-transcriptomics, the observation of changes in gene expression in multiple, interacting organisms simultaneously and opens new possibilities for metagenomic approaches studying genetic material sampled directly from the field. Coupling metagenomics and meta-transcriptomics to agricultural practices is likely to have a large impact on the understanding of biocontrol agents, plant defence induction, pathogens spread, spraying-regimes and integrated pest management.

Identification and quantification of proteins and metabolites in field-grown plants using “-omics” is a potential fast and cost-efficient way to discover key components in agronomically important traits and have been successfully applied to analyze genetic and/or environmental perturbations (Weckwerth, 2011). For example, a study employing rapid crop proteomic phenotyping of 12 tetraploid potato cultivars grown on three plots in two geographically separated fields misclassified less than 0.5% of the samples based on more than 4000 proteins despite high biological variability (Hoehenwarter et al., 2008, 2011). Obtaining global proteome samples from the field would be labor-intensive and require complicated fractionation steps impeding large-scale analysis. Consequently, targeted approaches are currently necessary. The secretome fraction containing apoplastic proteins is fast and relatively easy to isolate—even in the field—with a low level of contaminants of other cellular compartments (Alexandersson et al., 2013). It also constitutes the interface between intracellular processes and the environment. One way to overcome the complexity associated with protein quantification and identification by MS is to subject a set of proteins to selected reaction monitoring (SRM). SRM has high quantitative accuracy and can discriminate between protein isoforms in contrast to shotgun proteomics (Lehmann et al., 2008). A new workflow has been proposed which initially uses discovery proteomics in a controlled environment in order to choose amongst candidate proteins for SRM selection for analysis in samples obtained in the field (Jacoby et al., 2013). However, it remains to be seen whether it is more fruitful to choose candidates from more detailed proteomic studies directly in the field rather from a controlled environment since little is known about differences in field and laboratory proteomes (Montes et al., 2011). In field studies several factors that drive change of metabolites have been identified (Davies et al., 2010). In a recent long-term experiment for biodiversity research (http://www.the-jena-experiment.de/) metabolomics revealed a large variability in phenotypic plasticity of individual plant species (Scherling et al., 2010). Here, both gas and liquid chromatography coupled to MS was used to measure complementary sets of metabolites.

To explore the general effect of spatial variance or management methods, isolation of RNA is still preferable since it is relatively easy to sample if the specimen can be flash frozen and gives a global view of changes taking place, whereas analyzing metabolites currently might be more suitable to study variance in plant-tissue composition for desired properties, especially if the genes affecting the metabolites of key-interest are poorly characterized. Both proteomics and metabolomics are “closer to the phenotype” than RNA but more dependent on stability of different fractions or specific molecules. The abovementioned plant-secretome fraction, for example, might harbor more stable proteins and thus be more suitable for isolation in field conditions.

## Making sense of molecular data from the field

Conducting experiments in a multi-stress environment requires that spatial and temporal variance is taken into account and at least to some extent measured—this is probably why molecular biologists refrain from field experiments. Micro-climate and soil composition may in addition vary not only between fields but also over single plots. Sampling is also dependent on plant developmental stage and variance of biotic factors such as disease or symbiont load. Still, the importance of looking at multi-stress conditions has been emphasized and it is known that combined stresses can lead to distinct responses on the molecular level (Atkinson and Urwin, 2012).

The cumulative environment over the growth season will also affect mRNA, protein and metabolite levels. During grapevine berry development and ripening a large degree of transcriptome plasticity was observed when comparing growth years, agronomic practices and climate (Dal Santo et al., 2013). Interestingly, the levels of plasticity as well as the impact of external factors were dependent on the developmental stage of the berry. At the onset of ripening the seasonal climate had its greatest effect whereas the microenvironment and agronomic practices had only marginal impact. The strong effect of growth year then faded during later stages of berry ripening (i.e., the importance of developmentally-controlled genes increased).

Good field sampling calls for an understanding of the heterogeneity present at the molecular level and the pitfalls of failing to account for it. There is a danger that the results of an experiment will reflect the spatial patterns in the field rather than the experimental perturbation studied. This can lead to a lack of statistically meaningful results as heterogeneity overwhelms the effects of the perturbation, or, worse still, false results solely due to natural spatial patterns. Recently, different agricultural practices in vineyards were shown to influence the microbial composition, but also that dramatic spatial species heterogeneity exists within individual vineyards (Setati et al., 2012). This has profound implications on study-design and sampling strategies for field trials. Most of the design and sampling strategies presently used were developed well before the advent of techniques that point out the substantial molecular heterogeneity present in field conditions and consequently mainly revolve around optimisation for a small number of variables. In the “-omics” era, tens of thousands of variables are measured and a sampling design that is optimized for one of them may well impart bias for the many other variables of interest. As such, there is a need for new sampling strategies to be developed. For example, it is important to arrange that the same material can be split up in a representative fashion for different types of analysis or to analyse metabolites, proteins and transcripts from the same sample (Weckwerth et al., 2004; Valledor et al., 2014). In field conditions, light variation, temperature and relative humidity need to be recorded (and normalized to the extent possible) at least for sampling events and ideally over the entire growth season in order to capture accumulative effects. Wind strength, direction and precipitation are also factors to be considered. Regarding water status, leaf water potential is an example of a measurement that can be easily determined in the field by a pressure probe. Pesticide and fertilizing schemes are other important events to record and should be considered when planning sampling times.

Qualitative and quantitative estimation of biotic factors in the environment is difficult to perform and often overlooked. Normally, visual symptoms are recorded in order to estimate the presence of pathogens. However, these are indirect signs and field-captured images are difficult to quantify. Furthermore, similar symptoms can have different causal agents. Infection by viruses is yet another often neglected underlying factor. To this end metagenomics and meta-transcriptomics and the detection of viruses based on NGS could prove to be valuable. Fitness and yield outcomes as a consequence of cumulative combined factors are more easily recorded, but causality usually remains opaque. In general, high variance in the field due to a multi-stress and multi-organism environment calls for more elaborate experimental designs to ensure representative sampling.

## Field molecular data in a larger context

An important task in field-omics will be to relate “-omics” profiles to field observations and identify the external factors, such as stresses and management interventions, influencing molecular processes as well as ranking the importance of these factors. Such work will also provide relevant parameters to include in a laboratory setting to improve screening of field-relevant traits. Luckily, phenotyping and remote sensing of crops in the field is a rapidly developing area. For scientific applications several pitfalls still exist as has been pointed out by Walter et al. (2012). These are primarily associated with problems in retrieving good-quality images, mainly due to changes in light conditions.

The labor-intensive collecting and handling of field phenotype data has limited studies to measure a few traits effectively causing a “phenotyping bottleneck” (Furbank and Tester, 2011) that has become even more evident with increased availability of “-omics” techniques. New imaging pipelines facilitating high-throughput phenotyping (e.g., Hartmann et al., 2011) and novel frameworks and statistical methods for analysing and relating multiple phenotypic traits (Granier and Vile, 2014) are under development. Since most molecular measurements are destructive in nature, a key point in Field-omics will be to find non-intrusive characterization methods in order to minimize interventions. Knowledge and inspiration can be gained from post-harvest research including the use of tomography (Borisjuk et al., 2012).

Knowledge gained from laboratory experiments can be used to guide discovery when untangling the relationships between multiple environmental cues and plant responses. Literature mining is challenging, but descriptive tools such as the Gene Ontology are available and widely used (Ashburner et al., 2000). However, apart from these, other ontologies such as plant phenology (see http://www.ppodb.de/) remain underused and the practice needs to increase (overview of plant-related ontologies, Walls et al., 2012). However, usability should be improved. A standardization of experimental descriptions, reporting of meta-data and suitable repositories are also necessary to facilitate data comparisons. Ambitious efforts have been made, e.g., to store and share experimental set-ups (Hannemann et al., 2009) and examples for international networks and novel database approaches designed to analyse large-scale datasets are discussed in Weckwerth (2011).

One of the main challenges is still the ability to handle, analyze and visualize large-scale data so that biologists can take in and further advance biological understanding. Field studies are similar to clinical studies in that they face the effects of unknown orthogonal variables on the “-omics” data being collected and, as such, make statistical analysis quite difficult. Non-parametric agglomerative statistical methods such as gene set analysis (GSA) have been effective in analysing clinical data (Subramanian et al., 2005) and should be explored further in field-omics. One problem with GSA methods is incomplete set generation. However, network-driven set generation holds considerable promise in this regard (Jacobson and Emerton, 2012). Other exploratory data analysis methods such as principal components analysis (PCA) and partial least squares (PLS) can be helpful but tend to be overwhelmed by tens of thousands of variables and are solely linear in nature. Machine learning approaches, such as Random Forests and Support Vector Machines are also showing promise in handling these types of data. However, further work needs to be done with regards to statistical significance and variable selection. Network-based analysis is becoming increasingly popular for the analysis and visualization of “-omics” data and can seamlessly integrate different types of data with extant knowledge and thus merits further exploration (Wienkoop et al., 2008; Weckwerth, 2011).

## Future perspectives

At first glance, field-omics studies could seem to be restricted to empirical observations and data compilation. But in fact they require innovative experimental designs and the ability to come up with sound research questions in an outdoor setting—skills which need to be developed when it comes to capturing large-scale molecular biology data. Furthermore, as exemplified in the introduction, field studies have on several occasions led to novel discoveries as well as founded methodological frameworks.

A field-omics approach calls for closer collaborations between computational biologists, molecular biologists, plant physiologists and agronomists in order to bridge knowledge gaps and create economic drivers for comprehensive field studies. A mixture of concepts from plant breeding, microbiology, ecology and evolutionary biology are also important to create a theoretical framework and fruitful experimental set-ups. Notably, new agricultural field experiments might serve as models of how to develop ecological studies due to its intermediate position between a laboratory and nature in terms of numbers of controllable parameters (Table 1). Compared to ecosystems and clinical data, the field as an entity is a simplified and more controlled structure and thus has the power to connect mechanisms *in planta* with ecology. Gaining more insight in semi-controlled environments is also important to advance the understanding of greenhouse systems.

Biomarkers, which can be used as predictors (e.g., transcripts or metabolites) of biological processes or states, which are robust enough to function in the various environments experienced in field conditions, need to be identified. It is possible that more relevant ones or signatures of several (“-omics” profiles; Figure 1) could be identified in processing field rather than lab samples. For example, when sampling different types of vineyards avoiding infected berries for a genome-wide expression study, several pathogenesis-related (PR) proteins were found to be non-plastic, i.e., independent of external stress cues (Dal Santo et al., 2013). In cultivated potato, we have seen surprisingly few samples with high levels of PR proteins even with a high disease pressure. These proteins have been identified as classical biomarkers for stress-induction in the laboratory, but might be unsuitable as biomarkers in the field where they seem be absent or constitutively expressed.

Besides the potential use in breeding, “-omics” profiles could be important in precision agriculture and influence crop systems and farming practices. Several qPCR-based projects and patents use the expression of certain genes as biomarkers, e.g., to screen for good elicitors of plant defense in both laboratory and field (Brisset et al., 2013). Still, in medicine very few individual biomarkers have entered clinical practice and lessons should be learned to avoid false positives and improve predictions by using signatures of multiple biomarkers (Diamandis, 2012). Data integration strategies are of utmost importance in the future because each data level from molecular to field observation provides a different readout of the whole process. Furthermore, multivariate statistical analysis of an integrated data matrix has been shown to improve the selection and stability of biomarkers (Morgenthal et al., 2005; Wienkoop et al., 2008). Thus, there is a need to systematically store, compare and integrate different “-omics” data (see e.g., Colmsee et al., 2012). Finally, molecular field data should become integrated in crop performance models using a systems biology framework (Yin and Struik, 2008). This was illustrated by insect resistance to transgenic Bt-crops, where a more rapid development of resistance was forecast based on lab data than what actually occurred in the field (Tabashnik et al., 2003). However, to date there are few large-scale molecular data sets, for example phosphoproteome, RNA-seq or methylation data, from the field. These will be crucial to set a baseline for molecular field studies and learn about the differences in global molecular mechanisms compared to a laboratory setting.

### Conflict of interest statement

The authors declare that the research was conducted in the absence of any commercial or financial relationships that could be construed as a potential conflict of interest.
